# 392. Antiretroviral Prophylaxis to Prevent Perinatal HIV Infection in Medicaid-Insured Infants, 2009-2021

**DOI:** 10.1093/ofid/ofae631.127

**Published:** 2025-01-29

**Authors:** Mingyue Lu, Kengo Inagaki

**Affiliations:** University of Michigan, Ann Arbor, MI; University of Michigan, Ann Arbor, MI

## Abstract

**Background:**

All infants born to people living with human immunodeficiency virus (HIV) should receive postnatal antiretroviral prophylaxis. National policy surrounding perinatal HIV transmission has evolved over time, while real-life data on the effects of such policies are lacking.

HIV Infection within One Year of Life among Newborn Infants with or without Perinatal Antiretroviral Prophylaxis

**Figure:**
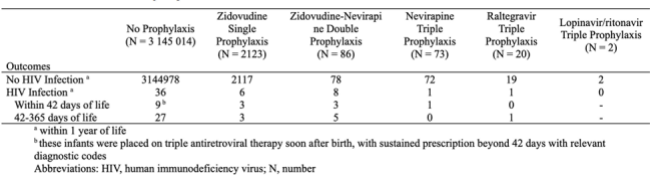

**Methods:**

We performed a population-based retrospective study using the MarketScan Multi-Sate Medicaid Database from 2009 to 2021. We identified antiretroviral drug use among infants and classified them into prophylactic and therapeutic use. A linear regression model was developed to identify the temporal trend of the use of the zidovudine single prophylaxis among infants receiving postnatal HIV prophylaxis.

**Results:**

We identified 3,147,318 newborn infants during the study period. Among them, 2304 received postnatal antiretroviral prophylaxis, with 2123 receiving zidovudine single prophylaxis in 2009, the rate dropped to 71.7% of such infants by 2021 as a result of the adoption of double and triple prophylactic regimens (P for trend < 0.001). Triple prophylaxis became more commonly used than double prophylaxis by 2018. During the study period, 52 infants had HIV infection within 1 year of life. Among them, 27 (51.9%) infants did not receive perinatal prophylaxis.

**Conclusion:**

Postnatal HIV prophylaxis strategies have evolved over time, reflecting the shifts in healthcare policy. Importantly, more than half of infants diagnosed with HIV during the first year of life did not receive perinatal prophylaxis, suggesting missed maternal infections. Healthcare policy is well-adopted in perinatal HIV infection prevention, but continued evaluation of its adequacy is necessary.

**Disclosures:**

**Kengo Inagaki, MD**, AstraZeneka: Grant/Research Support

